# The evolving role of truncal fascial plane blocks in non-surgical pain therapy: a narrative review

**DOI:** 10.3389/fmed.2025.1612201

**Published:** 2025-06-04

**Authors:** Yi Gu, Yifan Qin, Jin Wu

**Affiliations:** Department of Anesthesiology, Affiliated Hospital of Jiangsu University, Zhenjiang, China

**Keywords:** truncal fascial plane blocks, non-surgical pain management, erector spinae plane block, quadratus lumborum block, transversus abdominis plane block, serratus anterior plane block

## Abstract

Truncal fascial plane blocks (TFPBs), including erector spinae plane block (ESPB), quadratus lumborum block (QLB), transversus abdominis plane block (TAPB), and serratus anterior plane block (SAPB), are regional anesthesia techniques that achieves analgesia by injecting local anesthetics into a specific fascial planes of the trunk, which is primarily used for postoperative pain management or multimodal analgesia regimens. TFPBs reduce surgical site pain by blocking nerve conduction while decreasing reliance on systemic analgesics, such as opioids. This narrative review evaluates the analgesic efficacy and mechanisms of TFPB in non-surgical pain management, exploring their clinical value and future development prospects.

## Introduction

Fascial plane block (FPB), an emerging regional anesthesia technique, involves ultrasound-guided injection of local anesthetics into interfascial spaces to achieve nerve blockade (1). The mechanism of action appears to involve two pathways: direct local effects on nociceptive pathways and neural structures within the fascial plane or adjacent tissues, and systemic analgesic effects resulting from vascular absorption of the anesthetic agents (2). Beyond acute pain relief, fascial plane blocks (FPBs) have the potential to alleviate chronic pain through the inhibition of inflammatory responses. Additionally, FPBs may help mitigate neuroplastic changes associated with prolonged exposure to inflammatory stimuli or repeated opioid administration, thereby contributing to long-term pain modulation (3). On the basis of the mechanisms mentioned above, FPBs have demonstrated advantages in non-surgical pain management, as they offer broad sensory coverage, minimal motor impairment, lower systemic side effects, and potential anti-inflammatory benefits (4), making them a valuable tool for both acute and chronic pain management compared to other pain treatment methods (5). Studies have shown that among the various FPB techniques, truncal fascial plane blocks (TFPBs), including the erector spinae plane (ESP) block, quadratus lumborum block (QLB), transversus abdominis plane block (TAPB), and serratus anterior plane (SAP) block, have demonstrated promising results in reducing pain intensity and enhancing patient comfort in non-surgical pain management (6).

Despite their growing clinical application, several challenges remain, including variability in analgesic efficacy, differences in injection techniques, and the need for more robust clinical evidence in specific pain conditions. This review aimed to summarize the current evidence on TFPBs in non-surgical pain management, discuss their mechanisms of action, evaluate their clinical applications, and highlight future research directions.

## ESPB

Ultrasound-guided ESPB, originally described by Forero et al. (7) in 2016, involves the deposition of local anesthetics between the erector spinae muscle and the transverse process. Drug diffusion within the fascial plane enables multidirectional spread, with craniocaudal distribution patterns potentially extending to the paravertebral space. It can produce dual blockade of both dorsal and ventral spinal nerve rami, and sympathetic ganglia (8). This mechanism enables ESPB to achieve extensive sensory coverage spanning from cervical to lumbar regions. While conventional opioid-based analgesia demonstrates clinical efficacy, its application is limited by risks of respiratory depression, addiction and tolerance. In contrast, ESPB offers a safer and promising opioid-sparing alternative. Currently, ESPB has been widely utilized for perioperative analgesia for diverse surgeries (e.g., cardiothoracic, abdominal, spinal) (9). Meanwhile, ESPB demonstrates potential advantages in the management of non-surgical pain and may serve as an alternative therapeutic option to pharmacotherapy (10). The details of the included literature are shown in Table 1.

**Table 1:** Summary of the literature on ESPB for non-surgical pain.

Study	Type of article	Target disease	Number of patients	Intervention	Block characteristics	Main outcome measures	Results
Gopinath B et al. 2021 (12)	Case series	Severe acute pancreatitis	7	Bilateral ESPB	1% lidocaine 5 mg/kg, 0.5% bupivacaine 2 mg/kg, and dexamethasone 8 mg were injected at T7 level.	Consumption of opioids	Opioid cessation in 71% of cases (5/7) and substantial dose reduction in the remaining patients (2/7).
Chauhan G et al. 2022 (13)	Case report	Refractory chronic pancreatitis	1	Bilateral ESPB	0.25% bupivacaine and methylprednisolone 40 mg were injected at T6 level.	NRS	NRS 8/10 → 0/10
David SN et al. 2024 (14)	RCT	Acute hepatopancreaticobiliary pain	70	ESPB vs. morphine	0.2% ropivacaine was injected at T7 level.	NRS	The intervention group showed significantly lower NRS pain scores at 3, 5, and 10 h after ESPB.
Brewer J et al. 2022 (19)	Case report	Acute appendicitis	1	Unilateral ESPB	0.2% ropivacaine was injected at L1 level.	Pain score	The pain score decreased from 6/10 to 1/10.
Fujimura Júnior AY et al. 2025 (28)	Systematic review and meta-analysis	Postherpetic neuralgia	362	Clinical treatment combined with ESPB vs. clinical treatment alone	ESPB	Pain, PHN incidence, acetaminophen and pregabalin consumption	Pain, PHN confidence, acetaminophen and pregabalin consumption have all significantly improved.
Kumar A et al. 2020 (29)	Case report	Postherpetic neuralgia	1	Intermittent unilateral ESPB	0.25% bupivacaine plus triamcinolone acetonide was injected at L3 level.	NRS and Consumption of opioids	NRS 8/10 → 3/10 within 30 min and sustained 50% opioid reduction over 14 days.
Lin Z-M et al. 2021 (30)	RCT	Postherpetic neuralgia	50	ESPB vs. placebo subcutaneous injection	0.4% ropivacaine was injected at T7-L1 level every 24 h for 3 days.	VAS and McGill Pain Questionnaire	The ESPB group had significantly lower VAS score at rest and total score of short-form McGill Pain Questionnaire-2 (*p* = 0.046 and *p* = 0.001).
Hasoon J et al.2021 (33)	Case report	Post-mastectomy pain syndrome	1	Unilateral ESPB	0.25% bupivacaine plus 40 mg methylprednisolone was injected at T5 level.	NRS and Consumption of opioids	NRS 9 → 0; Opioids were completely discontinued after 3 months.
Hernandez N et al. 2020 (37)	Case report	Headache	4	Bilateral ESPB	0.25% bupivacaine and 2–4 mg dexamethasone were injected at T4 level.	Pain	Immediate relief of headache.
De Haan JB et al. 2019 (38)	Case report	Post-dural puncture headache	1	Unilateral ESPB	0.25% bupivacaine hydrochloride and 3 mg dexamethasone were injected at the T4 level	NRS	NRS 10 → 0
Lopes Dinis R et al. (39)	Case report	Lower limb pain	1	Continuous unilateral ESPB	0.5% ropivacaine was injected at the L3 level.	VAS	Severe pain (VAS 8/10) and resting moderate pain (VAS 5/10) → moving (VAS 2/10) and the absence of pain at rest (VAS 0/10).
Marcial et al. 2024 (42)	Case report	Phantom Limb Pain	1	Unilateral ESPB	0.25% bupivacaine, 1% lidocaine, 5 mcg/ mL epinephrine, and 40 mg methylprednisolone were injected at the right T3 level.	NRS	NRS 5/10 → 1/10
Ashworth H et al. 2022 (43)	Case report	Abdominal pain caused by metastatic colorectal cancer	1	Unilateral ESPB	0.5% bupivacaine was injected at T9 level.	Pain	Reported 1/10 pain.
Kalagara HK et al. 2019 (44)	Case report	Pain caused by non-small cell lung cancer	1	Continuous unilateral ESPB	0.5% ropivacaine was injected at T1 level with 1.	NRS	NRS 9/10 → 4/10
Altıparmak B et al. 2019 (45)	Case report	Breast cancer cases with osseous metastases refractory	2	Bilateral ESPB	0.25% bupivacaine, 2 mg dexamethasone, and normal saline were injected at T3/T6 levels.	NRS	NRS < 2/10
Sirohiya P et al. 2020 (46)	Case report	Pancoast tumor and refractory severe pain	1	Unilateral ESPB	0.375% ropivacaine and 40 mg triamcinolone acetonide were injected at T2-level.	NRS	NRS 7/10 → 1/10
Hasoon J et al. 2020 (47)	Case report	Chronic intercostal neuralgia	1	Bilateral ESPB	Lidocaine 1% -dexamethasone (4 mg/mL) mixture (8:2 ratio) was injected at T8 level.	Pain	Significant relief of pain

ESPB, erector spinae block; NRS, Numeric Rating Scale; VAS, Visual Analog Scale; RCT, randomized controlled trial.

### Acute and chronic visceral pain

The management of acute and chronic abdominal pain remains an ongoing challenge in clinical practice. A particular issue of persistent difficulty has been achieving effective analgesia while minimizing reliance on opioid-based pharmacotherapy, which is frequently constrained by dependence risks and adverse effect profiles. This therapeutic dilemma underscores the critical need for evidence-based non-opioid interventions and multimodal analgesic strategies to address both nociceptive and neuropathic pain components in abdominal pain.

### Pancreatitis

Pancreatitis, a prevalent gastrointestinal disorder, is characterized by severe abdominal pain as its cardinal symptom. Pain management in pancreatitis remains clinically challenging due to the complex interplay of visceral nociceptive signaling and neuroinflammatory pathways. Conventional first-line analgesics—including nonsteroidal anti-inflammatory drugs (NSAIDs), acetaminophen, and opioid agonists—frequently exhibit suboptimal efficacy or safety limitations in this population. These constraints are exacerbated by comorbidities such as renal insufficiency (contraindication for NSAIDs), hepatic impairment (limiting acetaminophen use), and the dependency risks associated with prolonged opioid administration. Furthermore, systemic inflammation in pancreatitis may alter drug pharmacokinetics, necessitating tailored dosing regimens to mitigate adverse outcomes (11). The visceral pain in pancreatitis primarily transmitted through sympathetic afferent fibers from T5-T10 spinal levels, originating from the celiac plexus and splanchnic nerve pathways. This neuroanatomical basis provides a rationale for thoracic ESPB. Anatomic studies suggest that ESPB performed at T4–T9 predominantly covers dermatomes T2–T10. Besides, the use of anti-inflammatory adjuvants (e.g., dexamethasone) may modulate central sensitization and neuroinflammatory pathways.

The emerging clinical studies support the analgesic potential of ESPB in acute and chronic pancreatic pain. In a case series by Gopinath et al. (12), ESPB performed at the T7 level using lidocaine-bupivacaine-dexamethasone mixture demonstrated significant opioid-sparing effects in severe acute pancreatitis (AP) patients. The intervention achieved complete opioid cessation in 71% of cases (5/7) and substantial dose reduction in the remaining patients (2/7). Chauhan et al. (13) reported a case of refractory chronic pancreatitis (pain scores NRS 8–10/10) that achieved complete pain resolution within 24 h following ESPB at T6 using a bupivacaine and methylprednisolone combination. The most compelling evidence comes from a randomized controlled trial by David et al. (14) (*N* = 70), which demonstrated superior analgesic efficacy of ESPB performed at T7 using ropivacaine compared to intravenous morphine. The intervention group showed significantly lower NRS pain scores at 3, 5, and 10 h after blockade, with no patient requiring rescue analgesia.

### Appendicitis

Appendicitis is one of the most prevalent causes of acute abdominal pain in both adults and children, typically presenting as right lower quadrant or periumbilical pain (15). In addition to surgical intervention, antibiotic therapy and effective analgesia are primary treatment modalities for appendicitis (16). Furthermore, visceral pain is generally more challenging to manage than somatic pain using opioids or anti-inflammatory drugs and often remains inadequately controlled (17). ESPB injected local anesthetics into the erector spinae, which then diffused through the deep fascial plane into the paravertebral space, thereby blocking the dorsal and ventral branches of the spinal nerves, as well as the communicating branches carrying sympathetic nerve fibers. Analgesia is provided by blocking the visceral and somatic fibers that innervate the spinal cord level (18). Brewer et al. (19) corroborated our viewpoint by reporting a case of acute appendicitis in which L1-level ESPB using 20 mL of 0.2% ropivacaine reduced pain scores from 6/10 to 1/10.

### Postherpetic neuralgia (PHN)

PHN, defined as pain lasting more than 3 months after the rash has healed, is one of the most intractable and common complications of herpes zoster (HZ) (20). The incidence of HZ demonstrates an age-dependent increase, with rising case numbers observed in recent years. PHN poses an increasing therapeutic challenge, as it often shows poor response to conventional therapies. Especially in refractory cases, Inappropriate use of analgesicsmay cause more harm than benefit (21). Anesthetic techniques such as paravertebral and epidural blocks have been shown to be effective in relieving pain and reducing the incidence of PHN (22, 23), but these techniques carry a high risk of complications (24, 25). Thus, developing effective and safe analgesic approaches is crucial for PHN management. ESPB has recently emerged as a safer and simpler alternative with fewer complications.

ESPB targets PHN through dual mechanisms: (1) local anesthetic blockade of thoracic/lumbar dorsal rami interrupts nociceptive transmission from affected dermatomes, and (2) corticosteroids (e.g., triamcinolone) suppress neuroinflammation by inhibiting cytokine-mediated glial activation and reducing ectopic neuronal discharges (26). Anatomic studies demonstrate that ESPB reliably achieves craniocaudal coverage of 3–8 dermatomes, rendering it particularly effective for multi-dermatomal HZ involvement (27). Clinical evidence supports the therapeutic efficacy of ESPB. Fujimura Júnior et al. (28) conducted a systematic review and meta-analysis to analyze the efficacy of ESPB in the treatment of pain associated with herpes zoster. Its target populations include patients with acute infections and those with PHN. They suggest that ESPB appears to relieve pain in patients with herpes zoster, with long-term benefits in the acute phase. In addition, ESPB reduced the need for analgesics within 12 weeks. Kumar et al. (29) reported a refractory PHN case (T11-S1 involvement) in an immunocompromised patient, where unilateral L3-level ESPB with 0.25% bupivacaine plus triamcinolone produced rapid NRS reduction from 8/10 to 3/10 within 30 min and sustained 50% opioid reduction over 14 days.

The randomized controlled trial by Lin et al. (*N* = 50) demonstrated that T7-L1 ESPB with 0.4% ropivacaine (25 mL per administration) three times daily reduced PHN incidence by 68% at 12 weeks compared to saline controls (30).

### Post-mastectomy pain syndrome (PMPS)

Chronic pain after mastectomy, then called intercostobrachial nerve entrapement syndrome, was first identified in a case series of patients who had undergone mastectomy in the 1970s. This is called postmastectomy Pain syndrome (PMPS), and the International Association for the Study of Pain (IASP) defines PMPS as persistent neuropathic pain that occurs shortly after mastectomy or lumpectomy, which is located on the anterior surface of the armpit of the chest, shoulder, or upper part of the arm (31). Regional block is currently considered to reduce the pain of PMPS, and many current studies have shown its great benefits as an adjuvant treatment to reduce the pain of PMPS and a variety of blocks have shown promise in PMPS (32). Hasoon et al. (33) reported a case where T5-level ESPB using 0.25% bupivacaine with 40 mg methylprednisolone produced immediate pain relief (NRS 9 → 0), with sustained 70% reduction at 1 month and complete opioid cessation by 3 months.

### Headache

Refractory headache refers to treatment-resistant headache disorders (34), including primary headaches such as migraine, tension-type headache, and cluster headache (35). Pharmacotherapy is constrained by drug interactions, contraindications, allergies, and inevitable adverse effects. Nerve blockade demonstrates efficacy in providing immediate, complete, and sustained relief for refractory primary headaches and associated autonomic symptoms (36).

Hernandez et al. (37) reported bilateral T4 ESPB provided immediate headache relief (NRS reduction: 7–10 to 0–2) in four cases, with 75% discharged within 24 h. De Haan et al. (38) documented complete resolution (NRS 10 → 0) of post-dural puncture headache (PDPH) in an obstetric patient following T4 ESPB with 0.25% bupivacaine and dexamethasone. Of note, the ESPB for PDPH can relieve headache symptoms but cannot correct the underlying pathological changes of PDPH; therefore, it should not be considered first-line therapy for this condition.

### Lower limb pain

Lopes Dinis et al. (39) described an 80-year-old male with septic shock secondary to right lower extremity cellulitis, presenting with movement-associated severe pain (VAS 8/10) and resting moderate pain (VAS 5/10).

An ultrasound-guided L3 ESPB was performed with perineural catheter placement using 30 mL of 0.5% ropivacaine. Pain intensity decreased significantly within 10 min, as evidenced by standardized pain assessment scales. During continuous tapered infusion therapy, the patient maintained stable pain control without requiring rescue analgesia.

### Phantom limb pain (PLP)

PLP describes persistent pain localized to or encompassing the entirety of an amputated limb. Etiologies include infectious, traumatic, residual limb-related, and postsurgical factors. Patients typically report diverse neuropathic sensations in the amputated region, including burning, stinging, soreness, tingling, and thermal dysesthesia with fluctuating intensity (40). While central mechanisms represent the primary pathogenesis of PLP, peripheral and psychological factors also contribute to its development (41). Patients frequently obtain inadequate pain relief from pharmacotherapy or develop intolerable adverse effects.

Marcial et al. (42) described a 23-year-old osteosarcoma patient who received right shoulder disarticulation for humeral tumor management. On the first day after surgery, the patient reported phantom limb numbness and pruritus refractory to conventional pharmacotherapy, with persistent severe pain. Ultrasound-guided ESPB was subsequently performed at the level of T3, resulting in immediate pain reduction (NRS 1/10) without complications and progressive opioid requirement reduction over 72 h.

### Cancer pain

Cancer-related pain significantly impairs patients’ quality of life and prognosis. Conventional pharmacotherapy is often limited by adverse effects and dependency risks.

Ashworth et al. (43) documented a 54-year-old female with metastatic colorectal carcinoma presenting with refractory severe abdominal pain despite multimodal analgesic therapy. ESPB was performed at the T9 transverse process level using 3–5 mL of 0.5% bupivacaine, achieving complete analgesia within 30 min. Kalagara et al. (44) described a 55-year-old male with non-small cell lung cancer whose right Pancoast tumor invaded the brachial plexus and chest wall (including first and second ribs), resulting in refractory chronic pain in the right upper extremity, neck, and chest wall despite multimodal pharmacotherapy. ESPB was performed at the T1 level with 15 mL of 0.5% ropivacaine, followed by continuous infusion via an indwelling catheter. While the patient experienced immediate pain relief post-block, severe pain recurred following catheter removal. Başak Altıparmak et al. (45) described two breast cancer cases with osseous metastases refractory to conventional pharmacotherapy. Bilateral ESPB at T3/T6 levels was performed using 10 mL of 0.25% bupivacaine, 2 mg dexamethasone, and 5 mL normal saline. The first patient achieved sensory blockade spanning C8-T10 dermatomes, while the second showed T1-T9 coverage. Both maintained NRS < 2/10 without rescue analgesics for 24 h post-procedure. Prashant Sirohiya et al. (46) described a 42-year-old male with right Pancoast tumor and refractory severe pain (NRS 7/10 for 5 months) who underwent T2-level ESPB using 20 mL of 0.375% ropivacaine and 40 mg triamcinolone acetonide, achieving significant analgesia (NRS 1/10) within minutes.

### Chronic intercostal neuralgia

Jamal Hasoon et al. (47) performed bilateral ESPB in a 45-year-old male with refractory chronic intercostal neuralgia (average NRS 7/10) using 10 mL of a lidocaine 1%-dexamethasone (4 mg/mL) mixture (8:2 ratio). Following significant initial pain relief, the contralateral block was repeated, resulting in sustained bilateral analgesia for 10 months with occasional NSAID requirements.

## QLB

QLB originated from the posterior “non-breakthrough” TAPB proposed by Blanco in 2007 (48). While its precise mechanism remains unclear, current evidence suggests two potential pathways. Local anesthetic spread from the thoracolumbar fascia (TLF) to the paravertebral space may produce multisegmental somatic and sympathetic blockade. However, recent studies indicate minimal actual paravertebral diffusion. The TLF contains mechanoreceptors, nociceptors, and sympathetic fibers that may be directly modulated by the local anesthetic (49). Clinical studies have demonstrated QLB’s efficacy in both acute and chronic pain management (50). The details of the included literature are shown in Table 2.

**Table 2:** Summary of the literature on QLB for non-surgical pain.

Study	Type of article	Target disease	Number of patients	Intervention	Block characteristics	Main outcome measures	Results
Zhou et al. 2022 (52)	Case report	Primary dysmenorrhea	1	Bilateral anterior QLB	0.375% ropivacaine.	Pain	The pain was significantly relieved and no analgesic measures were required after 48 h.
Gonçalves J et al. 2021 (53)	Case report	Pain due to superior mesenteric vein thrombosis	1	Bilateral posterior QLB	0.375% ropivacaine.	Pain	The patient’s pain was significantly improved.
Fernandez MT et al. 2023 (55)	Case series	Chronic hip pain	20	Single-shot posterior QLB	0.25% levobupivacaine and dexamethasone 4 mg.	NRS and WOMAC mean value	NRS mean value 8 → 3; WOMAC mean value 72 → 37.
Carvalho R et al. 2017 (58)	Case report	Chronic pain after hernia repair	1	Bilateral posterior QLB	0.2% ropivacaine and methylprednisolone 20 mg.	VAS	VAS:8/10and9/10 → 0/10(in 60 mins); VAS 2/10 at rest and 6/10 in motion(at the first month of the procedure); VA S 3–4/10 at rest and in motion(at the 6 months of the procedure).
Barreto Silva A et al. 2023 (59)	Retrospective observational study	Myofascial pain syndrome of the quadratus lumborum	90	Posterior QLB	0.25% levobupivacaine and 40 mg triamcinolone	Pain	Pain intensity (5-point NRS) showed significant improvement at all follow-up intervals (72 h, 1, 3, and 6 months) compared to baseline.

QLB, quadratus lumborum block; NRS, Numeric Rating Scale; VAS, Visual Analog Scale; WOMAC, the Western Ontario and McMaster Universities Arthritis Index.

### Primary dysmenorrhea

Primary dysmenorrhea refers to pain that occurs during the menstrual cycle without a clear cause. It is one of the most common causes of pelvic pain in women. Dysmenorrhea can negatively affect a woman’s quality of life and interfere with daily activities (51).

Zhou et al. reported a patient suffered from severe pain caused by primary dysmenorrhea, and oral non-steroidal anti-inflammatory drugs were ineffective. The pain was significantly relieved after a single bilateral anterior QLB with 20 mL of 0.375% ropivacaine, and no other analgesic measures were needed within 48 h after block (52).

### Pain due to superior mesenteric vein thrombosis

Superior mesenteric vein thrombosis caused acute diffuse abdominal pain, which was ineffective with oral and intravenous analgesics. Gonçalves et al. reported a case in which bilateral posterior QLB with 20 mL of 0.375% ropivacaine could effectively manage this pain coming from exclusive visceral source (53).

### Chronic hip pain

Chronic hip pain secondary to femoral head necrosis, osteoarthritis, or impingement syndrome significantly impairs functional outcomes and complicates clinical management (54).

Fernandez et al. (55) conducted a study of 20 hospitalized patients with hip pain secondary to joint injury. Participants received an L3-L4 QLB using 20 mL of 0.25% levobupivacaine with 4 mg dexamethasone. Follow-up assessments revealed clinically significant improvements in both pain scores and functional outcomes. Notably, 50% of patients (*n* = 10) maintained adequate analgesia without adjunct interventions throughout the 12-month follow-up period.

### Chronic pain after hernia repair

Hernia surgery and chronic pain specialists recommend the definition of chronic pain after hernia repair as pain that persists for at least 6 months after surgery (56). The reason for this long time is that the inflammation around the mesh persists after 3 months and that it is possible for some patients to improve substantially 3 to 6 months after surgery (57).

Carvalho et al. (58) reported a 61-year-old patient with chronic post-herniorrhaphy abdominal wall pain who underwent bilateral posterior QLB using 25 mL of 0.2% ropivacaine and 20 mg methylprednisolone per side. The intervention provided immediate symptom relief, with sustained pain reduction maintained at 6-month follow-up.

### Myofascial pain syndrome of the quadratus lumborum

Barreto Silva et al. (59) conducted a retrospective observational study evaluating levobupivacaine-triamcinolone QLB in 90 patients with quadratus lumborum myofascial pain syndrome. A mixture of 10 mL of 0.25% levobupivacaine and 40 mg triamcinolone was injected between the erector spinae muscle and the posterior surface of the quadratus lumborum muscle. Pain intensity (5-point NRS) showed significant improvement at all follow-up intervals (72 h, 1, 3, and 6 months) compared to baseline, though opioid requirements remained unchanged.

## TAPB

Rafi (60) first described the TAPB, which involves injecting local anesthetic into the fascial plane of the abdominal muscles to achieve analgesia by blocking sensory nerve transmission. The TAPB disrupts the sensory innervation of the abdominal wall, which originates from the anterior rami of the thoracolumbar nerves (61). These sensory nerves reside within the interfascial plane between the internal oblique muscle and the transversus abdominis muscle (62). The details of the included literature are shown in Table 3.

**Table 3:** Summary of the literature on TAPB for non-surgical pain.

Study	Type of article	Target disease	Number of patients	Intervention	Block characteristics	Main outcome measures	Results
Sahoo RK et al.2015 (64)	case report	Chronic abdominal wall pain	20	TAPB	Methylprednisolone 20 mg and 0.375% ropivacaine.	Pain	Pain relief

TAPB, transversus abdominis plane block.

### Chronic abdominal wall pain (CAWP)

CAWP is one of the most common causes of chronic abdominal pain (CAP), which is often misdiagnosed and delayed in clinical treatment. The most important cause of CAWP is compression of the cutaneous nerve at the lateral margin of the rectus abdominis muscle, known as anterior cutaneous nerve entrapment syndrome (ACNES) (63).

Sahoo et al. (64) reported two cases of TAPB analgesia in patients with ACNES. Both patients were diagnosed with CAWP following their second cesarean delivery. After pharmacological therapy failed to achieve significant pain relief, a TAPB was performed. Subsequently, both patients demonstrated substantial pain alleviation, and no exacerbation of pain was reported during subsequent follow-up evaluations.

## SAPB

SAPB was originally proposed by Blanco et al. in 2013 for postoperative analgesia in breast cancer (65). With the deepening of research, the application of SAPB in the treatment of refractory pain has gradually increased, especially in the pain management after thoracic surgery. SAPB provides analgesia to the anterolateral chest wall by blocking the lateral cutaneous branch of the intercostal nerve, the long thoracic nerve and the thoracic dorsal nerve. The block range is roughly between T2 and T9, but the specific effect is affected by drug volume, injection site and other factors. As an emerging regional block technique, SAPB boasts high positioning accuracy, high success rates, and favorable safety profile with minimal complications (66). The details of the included literature are shown in Table 4.

**Table 4:** Summary of the literature on SAPB for non-surgical pain.

Study	Type of article	Target disease	Number of patients	Intervention	Block characteristics	Main outcome measures	Results
Semyonov M et al.2021 (68)	Retrospective observational study	Post-thoracotomy pain syndrome	91	SAPB vs. Drug analgesia	SAPB	Pain	Significant improvement in pain intensity.

SAPB, serratus anterior plane block.

### Post-thoracotomy pain syndrome (PTP)

PTP is a serious complication of thoracic surgery, which occurs at least 2 months after thoracotomy at the incision scar and is characterized by persistent or recurrent pain. The exact mechanism of PTPS pathogenesis is unclear, but there are some cumulative evidences pointing that it is a combination of neuropathic and nonneuropathic (myofascial) pain. Conventional analgesic approaches often prove inadequate, significantly compromising patients’ quality of life and postoperative recovery outcomes (67).

Semyonov et al. (68) retrospectively analyzed 91 patients with PTPS, comparing ultrasound-guided SAPB with conventional opioid/NSAID therapy. At 6-month follow-up, the SAPB group showed significant reductions in burning/stabbing or shooting, electric-shock-like, pressure-like pain, and overall pain intensity. Additionally, patients in the SAPB group reported marked alleviation of pain localized to the superior, inferior, and posterior thoracic anatomical regions.

## Conclusion

In conclusion, this review focuses on the application of four specific TFPBs—ESPB, QLB, TAPB, and SAPB—in managing a variety of non-surgical pain conditions. The existing evidence, including case reports, retrospective studies, meta-analyses, and randomized controlled trials, suggests their potential efficacy specifically in the acute, chronic, and refractory pain discussed in this review. However, more randomized controlled trials or multicenter clinical studies are needed to evaluate the efficacy of different TFPB approaches, optimize the drug regimen, and clarify the long-term outcomes and underlying mechanisms, thereby enhancing its clinical value in managing non-surgical pain.
